# Neonatal Screening on Tandem Mass Spectrometry as a Powerful Tool for the Reassessment of the Prevalence of Underestimated Diseases in Newborns and Their Family Members: A Focus on Short Chain Acyl-CoA Dehydrogenase Deficiency

**DOI:** 10.3390/ijns6030058

**Published:** 2020-07-28

**Authors:** MariaAnna Messina, Alessia Arena, Agata Fiumara, Riccardo Iacobacci, Concetta Meli, Federica Raudino

**Affiliations:** Referral Center for Inherited Metabolic Diseases, Pediatric Clinical, AOU Policlinico-VE, Via Santa Sofia 78, 95123 Catania, Italy; alessia.arena@gmail.com (A.A.); agafiu@virgilio.it (A.F.); riki713@hotmail.it (R.I.); cmeli@policlinico.unict.it (C.M.); federica.raudino@tiscali.it (F.R.)

**Keywords:** newborn screening, tandem mass, short chain acyl-CoA dehydrogenase deficiency, prevalence

## Abstract

Early detection of disabling diseases, prior to clinical manifestations, is the primary goal of newborn screening (NS). Indeed, the required number of core and secondary conditions selected for screening panels is increasing in many countries. Furthermore, newborn screening can lead to diagnosis of maternal diseases such as vitamin B12 deficiency or 3-MethylcrotonylCoA-carboxylase deficiency (3MCC). NS became mandatory in Sicily in December 2017. Here we report NS data collected between December 2017 and April 2020. Our results show that tandem mass spectrometry is a powerful tool for discovery of underestimated disease in newborns and their family members. Our panel included short chain acyl-CoA dehydrogenase deficiency (SCADD). Here, we report that results of our investigation led to reassessment of SCADD prevalence in our population. The infant and adult patients diagnosed in our study had previously not shown overt symptoms.

## 1. Introduction

In Italy, expanded newborn screening became mandatory, in all 20 Italian regions, in 2016, following passage and enactment of law N.167. The following year, the autonomous region of Sicily adopted the law and designated two screening centers, one for each, the east and west sides of the island. On the east side, in Catania, is University Hospital Policlinico. Hospital Civico is in Palermo. Each has expected catchments of about 22,000 births per year. The full panel of required disorders includes 44 metabolic diseases performed by tandem mass spectrometry (Table 1).

Previous workers have shown that primary markers for some pathologies are useful for making the differential diagnosis between two or more diseases. In addition, other markers revealed by NS screening, aid in diagnosis of maternal metabolic disorders. These include vitamin B12 deficiency, 3-MCC deficiency, and carnitine uptake deficiency (CUD) [1,2,3,4,5,6,7].

However, our work reveals that abnormal values of specific markers in a newborn’s metabolic profile can lead to incidental discovery of corresponding diseases in family members. Thus, we investigated eleven cases of short chain acyl-CoA dehydrogenase deficiency (SCADD), which occurred in six index cases and five family members.

We focus on SCADD for two reasons. First, there may be a decrease of primary marker signal in the second spot sample, caused by, either, normalization or significant decrease of primary marker in the second spot of positive babies. Second, there may be a loss of SCADD patients without a correct investigation. Here, missing data causes errors in determination of disease prevalence.

Incidence and genetic characteristics of inherited metabolic disorders (IMDs) vary considerably between countries and populations [8,9]. Thus, testing errors could result in failure to correctly identify and update information regarding frequency of disorders and their distribution.

In that context, next generation sequencing (NGS) plays a crucial role. The application of NGS technologies enables the analysis of a large group of targeted genes involved in numerous inherited metabolic diseases. For this reason, NGS represent the best choice for identification of IMDs following expanded newborn screening program, in order to correctly predict the prevalence of inherited metabolic disorders.

Thus, we present screening data for newborns and affected individuals, confirmed by genetic analysis, of primary markers for SCADD, in Sicily, from December 2017 to April 2020.

## 2. Materials and Methods

Blood samples, collected between 36 and 72 h of life, are spotted on a Whatman 903TM filter paper (Eastern Business Forms, Greenville, SC, USA).

Each assay was carried out using the Neobase2 TM Non-derivatized MSMS kit (Wallac Oy, Turku, Finland), according to the manufacturer’s instructions. Briefly, using an automatic puncher (Perkin-Elmer Panthera Puncher, Waltham, MA, USA) filter paper disks (3.2 mm in diameter) were punched out from the dried blood spot (DBS) into the wells of a microplate and 125 μL of a working solution were added into each well. Each plate was shaken in the incubator/shaker (TriNest incubator, Wallac Oy, Turku, Finland) at 30 °C for 30 min at speed of 750 rpm, then 100 μL from the content of each well were transferred to another microplate to avoid the presence of the paper disk during the injection inside the mass spectrometer. The electrospray ionization-tandem mass spectrometry (ESI-MS/MS) analyses were performed on a 210 MD QSight™ spectrometer (Perkin Elmer) equipped with a triple quadrupole. Each measurement was performed in flow injection mode using the QSight™ HC Autosampler MD (Perkin Elmer) and QSight™ binary pump (Perkin Elmer), volume of injection 10 μL, flow rate 0.02 mL/min. The MS parameters were set up as follows: capillary voltage 5 kV, source temperature 175 °C, drying gas 105 L/h and nebulizer gas 130 L/h. The analytical measurements were performed in the multiple reaction monitoring mode (MRM), using stable isotope internal standards for quantitative analysis. The cut-off criteria were set up using the 99‰ of the healthy population.

Urinary organic acids analyses were performed with a mass selective detector Agilent 5977B coupled to a 7890B gas chromatograph (GC/MS, Agilent Technologies, Santa Clara, CA, USA) using a HP-5ms capillary column (30 m × 0.25 μm × 250 μm), as described in literature [10]. Genetic analyses were performed on MiniSeq (Illumina, San Diego, CA, USA) with the Paired-End 150 bp protocol. The sequencing was preceded by selective enrichment using an amplicon-based strategy (Ampliseq for Illumina, Illumina). Total coverage of the coding gene was ensured by Sanger sequencing.

## 3. Results

During the NS mandatory program, until April 2020, we screened 50,521 babies, of which 6233 were preterm babies (12.3%). We recalled 364 babies corresponding to a recall rate of 0.69. Twenty-five newborns out of 364 recalled newborns were confirmed for inherited metabolic disorders (Table 2) using molecular techniques.

As reported in Table 2 phenylketonuric\hyperphenylalaninemic babies are the most prevalent (prevalence 1:5052), followed by babies affected by SCADD (prevalence 1:8420). The last Italian report drawn up by the Italian Society for Inherited Metabolic Disorders and Newborn Screening disclose a prevalence for SCADD of 1:14749, so our data seem to indicate a higher prevalence of the disease in our population. In particular, 48 babies were recalled for butyrylcarnitine (C4) values higher than cut-off (0.62 M), and therefore they represent 13.2% of the total recalled babies.

Our studies revealed eleven cases of SCADD, six index cases and five family members (Table 3, Table 4).

The results revealed that all participating newborns carry the c.625G>A allele. A variant common in other populations is c.511C>T, but it was not present in our population. We identified two babies who were compound heterozygous, the first one for the variants c.625G>A, c.320G>A (case 1) and the second one for the variants c.625G>A, c.136C>T (case 2). Only one baby resulted homozygous for the variant c.625G>A (case 3). Two babies were homozygous for this same variant and heterozygous for the known mutation c.1147C>T (case 4 and 5), while the final newborn was homozygous for the variant c.625G>A and heterozygous for the deletion c.366_367delCT (case 6).

Further, as reported in Table 4 we found SCADD in five adult patients: the sister and the father of the case 4 (case a and b), the father of the case 2 (case c), the mother of the case 6 (case d) and the mother of the case 5 (case e). All of them were positive for EMA on their urine organic acid profile.

## 4. Discussion

### 4.1. Correlation of the Genotype to C4 Levels in DBS and EMA Excretion

Six of 48 babies recalled for C4 variations were confirmed by molecular techniques.

Compared to initial screening values, C4 values of a second DBS, decreased in all six newborns. As widely reported by other labs, the amount of time between sample collections affects the level of acylcarnitine. In fact, as with very long chain acyl-CoA dehydrogenase deficiency (VLCADD), C4 levels in some individuals, overall homozygous with the c.625 G>A variant, in a second DBS, collected after five days of life, have been reported to fall below the cut-off value [11,12,13]. Our newborn recalls were, on average, performed eight days after collection of the first sample. This suggests that time between collection of samples contributes to the low values observed in the second DBS. Van Maldegem attributes this feature to the caloric intake of the babies after the first days of life when, on the contrary, they could be in a catabolic condition with an enhanced-oxidation. Roe, however, tested the relative activities of human cDNA for methyl-butyryl-CoA dehydrogenase (MBCD) in E. coli and found that the enzyme prefers S-2-methylbutyryl-CoA but showed a 55% increase in activity with butyryl-CoA compared to S-2-methylbutyryl-CoA. He concludes that the human enzyme apparently uses both straight-chain and branched-chain substrates efficiently rather than being limited to only the valine and isoleucine pathways [14].

In light of these results we can assume that during the period of metabolic adjustment in the first days of life, which characterize the transition between caloric intake and the catabolic condition of subsequent days, MBCD may contribute to disposal of C4 which accumulates in SCADD-affected babies. Indeed, the n-butyryl-CoA could be catabolized by MBCD and this can contribute to the decrease, in a second DBS, of the C4 carnitine signal value.

Our data show that C4 values returned to high levels in two infants. Both were homozygous for c.625G>A, and heterozygous for variant 1147C>T. The other four babies continued to have reduced values, lower than cut-off, of C4.

Furthermore, ethylmalonic acid (EMA) was excreted in the babies’ urine. The urinary organic acid profile was performed concurrently with the second DBS analyses of acylcarnitine. As reported in Table 2, increased EMA excretion occurred in the babies carrying the heterozygous c.1147C>T in homozygosity with c.625G>A.

Gregersen identified the variant c.1147C>T as a severe disease-causing SCADD. He also reported occurrence of the mutation in heterozygous form, with c.625G>A and c.511C>T, in three patients with elevated EMA excretion [15]. Our findings support the Gregersen data.

Our data further suggest that the time period between collection of the two DBS, in concert with the infant’s feeding status and inherited genetic mutations, casts doubt on the reliability of a single evaluation of C4 retest samples. Therefore, we recommend testing of multiple samples and several analyses.

Data from adult patients identified as a consequence of NS are shown in Table 4.

Among adult patients, cases a, b and c differ from cases d and e by the homozygousity of variant c.625G>A. In the first three patients, we found high excretion of EMA in urine and high values of C4 on DBS, comparable to that of cases 4 and 5, who carried the same genetic mutations. For similar reasons, case c, a compound heterozygote for the same mutations, measured very high EMA excretion and very high values of C4 on DBS. Furthermore, case 2 excreted EMA only after several days of retesting. The mutation c.136C>T is described as a rare inactivating mutation for which computer-assisted analysis predicts significant changes in the secondary structure of the NH2-terminal region of p.SCAD [16,17]. This might explain why carriers of this allele have the highest C4 value among all the cases identified as homozygous for c.625G>A and heterozygous for other pathological variants.

### 4.2. Correlation of the C4 to Other Markers in DBS

All newborns recalled for impairment of C4 values, had normal values of C0 and C2. Thus, the C4/C2 ratio and the evaluation of free carnitine, was not informative, even if C0 values of cases 4, 5 and 6 were not significantly higher than the lower cut-off (8.9 M).

Recently, Wang and coworkers proposed a new ratio, C4/C5DC+C6OH, as an independent indicator for identification of SCADD in NS. They concluded that research based on large sample sizes and data from multicenters would be required [18]. However, in our population, despite the value of the ratio is always higher than the cut-off (cut-off value = 6) into the whole of recalled babies, there is no significative difference between the value of confirmed babies and false positive. In fact, negative newborns showed values much lower than cut-off. Their values run to around 2 (media = 2.78, median = 2.55), while the confirmed babies and false positive showed similar values (respectively, media = 8.92, median = 8.62 and media = 9.10, median = 9.59).

### 4.3. Significance and Ethical Implication of SCADD Screening

The mutations c.511C>T and c.625G>A have been widely described in exons 5 and 6 of the SCAD gene in European populations and the population of the State of California, U.S.A. [11,19,20,21,22]. Although they modify the structural and catalytic properties of the SCAD protein, these variants are believed to confer susceptibility for developing SCADD [12].

Indeed, it has been proposed that these polymorphisms, when homozygous or heterozygous with another pathogenic mutation, increase susceptibility to symptoms under particular conditions, such as fever [12,15,23]. However, the clinical heterogeneity in SCADD is varies widely. Clinical signs and symptoms range from asymptomatic to symptomatic with hypotonia, failure to thrive, developmental delay, retardation or epilepsy. Moreover, it’s still not clear why only certain individuals carrying these variants develop clinically relevant disease.

Despite SCADD, diagnosed by newborn screening, presents largely as a benign condition and seems to have no clinical relevance, it is included as a secondary condition on the list of the Recommended Uniform Screening Panel (RUSP) of the Advisory Committee on Heritable Disorders of Newborns and Children (ACHDNC).

Kim Y-M reports that, although most patients with SCADD show either mild clinical symptoms or none at all, SCADD is not a silent disease. Concern abounds for not only patients’ long-term neurodevelopmental status and metabolic crises, but also for understanding disease pathogenesis and effective treatments [24]. Similarly, Lysiova comments on the importance of early detection this disorder before a severe and/or possible life-threatening manifestation [19].

SCADD remains a controversial disease from an ethical point of view. However, different authors agree that it is difficult to conclude if patients with mutations are more prone to symptoms or if homozygosity for ACADS common variants can lead to a metabolic decompensation when combined with other stressors. Prospective studies with longer follow-up durations are needed with careful monitoring of biochemical values, clinical outcome and neurodevelopment [21]. As well as molecular analysis, which also evaluates the role of uncommon and often uncharacterized variants, along with in silico functional studies, provides physicians with essential information to manage patients diagnosed with SCADD [22].

For this reason, in our opinion, the evaluation of these variants, especially when they are heterozygous with another pathogenic mutation, remains of interest, in order to better understand their implications in SCADD and correlation to C4 values on DBS.

The diagnosed babies range from nine months to 1 year and half old. Up to now, they are still asymptomatic. However, low fat and high carbohydrate diet was widely recommended.

During the metabolic and genetic counselling, the adult patients were informed about the significance of SCADD as well as the importance to avoid fasting and the possibility of riboflavin and L-carnitine as treatments.

### 4.4. Limitation of the Study

In our study, the evaluation of free carnitine and secondary ratios was not informative.

On these grounds, considering the potential importance of adding a second tier test, a retrospective study on correlation between the biochemical data and genetic analysis as well as the values of ethylmalonic acid on DBS would be desirable, and is our short-term goal.

Although some adult patients described their own muscle weakness and needing to eat small, frequent meals, we do not have medical examination findings of muscle weakness or further laboratory data. Until now, none of our patients shows severe clinical signs. So, a clear genotype-phenotype correlation in SCADD has not been observed.

## 5. Conclusions

Neonatal screening by tandem mass spectrometry is a powerful tool for discovery of underestimated diseases in newborns and their family members. For the first time, through newborn screening, we identified two fathers affected by SCADD. Our data showed a prevalence for SCADD of 1:8420, relatives excluded, or 1:4593, relatives included. Despite that babies with SCADD identified through newborn screening have been well at the time of diagnosis and most have remained asymptomatic, we believe it is important to know the correct prevalence of the disease. Determining the birth prevalence of the disorders is a first step to better understanding the short- and long-term disease management. These data may also significant for the public health, from an epidemiological point of view, in order to know how the disorder impacts a population. Through genetic analysis we confirm the variant c.625G>A in all patients. We found a considerable increase of urinary EMA and DBS C4 in the patients carrying the homozygous c.625G>A in heterozygosity with another pathogenetic variant. However, infant and adult patients diagnosed in our study have thus far not shown overt symptoms. Finally, although the value of C4 on second DBS may fall below the cut-off value, we recommend the collection and testing of additional samples beyond the immediate newborn period.

## Figures and Tables

**Table 1:** Full panel of disorders performed by tandem mass spectrometry.

Disorder	Abbreviation	Marker
Phenylketonuria	PKU	Phe
Benign hyperphenylalaninemia	H-PHE	Phe
Biopterin defect in cofactor biosynthesis	BIOPT (BS)	Phe
Biopterin defect in cofactor regeneration	BIOPT (REG)	Phe
Tyrosinemia type I	TYR I	Tyr
Tyrosinemia type II	TYR II	Tyr
Maple syrup urine disease (MSUD)	MSUD	Val; Ile + Leu
Homocystinuria (CBS deficiency)	HCY	Met
Homocystinuria (severe MTHFR deficiency)	MTHFR	Met
Glutaric acidemia type I	GA I	C5-DC
Isovaleric acidemia	IVA	C5
Beta-ketothiolase deficiency	BKT	C5:1; C5-OH
3-hydroxy-3-methylglutaric acidemia	HMG	C5-OH; C6-DC
Propionic acidemia	PA	C3
Methylmalonic acidemia (Mut)	MUT	C3
Methylmalonic acidemia (A)	CbI A	C3
Methylmalonic acidemia (B)	CbI B	C3
Methylmalonic acidemia with homocystinuria (CbI C)	CbI C	C3
Methylmalonic acidemia with homocystinuria (CbI D)	CbI D	C3
2 methylbutyryl CoA dehydrogenase deficiency	2MBG	C5
Malonic acidemia	MAL	C3-DC
Multiple carboxylase deficiency	MCD	C5-OH
Citrullinemia type I	CIT	Cit
Citrullinemia type II	CIT II	Cit
Argininosuccinic acidemia	ASA	Cit
Carnitine transporter defect	CUD	C0
Carnitine palmitoyl transferase 1A deficiency	CPT I	C0
Carnitine-acylcarnitine translocase deficiency	CACT	C16; C18:2; C18:1; C18
Carnitine palmitoyltransferase II deficiency	CPT II	C16; C18:2; C18:1; C18
Long chain 3-hydroxyacyl-CoA dehydrogenase deficiency	VLCAD	C14:1; C14:2; C14
Trifunctional protein deficiency	TFP	C16:1-OH C16-OH; C18:1-OH; C18-OH
Long chain 3-hydroxyacyl-CoA dehydrogenase deficiency	LCHAD	C16:1-OH; C16-OH; C18:1-OH; C18-OH
Medium chain acyl-CoA dehydrogenase deficiency	MCAD	C6; C8; C10:1; C10
Medium- and short-chain-3-hydroxyacyl-CoA dehydrogenase deficiency	M/SCHAD	C4-OH
Glutaric acidemia type II	GA2/MADD	C4-C18 saturated, unsaturated
Tyrosinemia type III	TYR III	Tyr
Glycine N-methyltransferase deficiency	GNMT	Met
methionine adenosyltransferase deficiency	MAT	Met
S-adenosylhomocysteine hydrolase deficiency	SAHH	Met
3-methylglutaconic acidemia	3MGCA	C5-OH
3-methylcrotonyl-CoA carboxylase deficiency	3MCC	C5-OH
2-methyl-3-hydroxybutyryl-CoA dehydrogenase deficiency	2M3HBA	C5:1; C5-OH
Isobutyryl-CoA dehydrogenase deficiency	IBG	C4
Short-chain acyl-CoA dehydrogenase deficiency	SCAD	C4

Phe = p henylalanine; Tyr = tyrosine; Val = valine, Ile = isoleucine; Leu = leucine; Met = methionine; C5-DC = glutarylcarnitine; C5 = isovaleryl/2-methyl-butyryl-carnitine; C5:1 = tiglyly/3-methylcrotonyl-carnitine; C5-OH = 3-hydroxyisovalerylcarnitine; C6-DC = adipylcarnitine; C3 = propionylcarnitine; C3-DC = malonylcarnitine; Cit = citrulline; C0 = free carnitine; C16 = palmitoylcarnitine; C18:2 = linoleylcarnitine; C18:1 = oleylcarnitine; C18 = stearoylcarnitine; C14:1 = tetradecenoylcarnitine; C14:2 = tetradecadienoylcarnitine; C14 = myristoylcarnitine; C16:1-OH = 3-hydroxyhexadecenoylcarnitine; C16-OH = 3-hydroxyhexadecanoylcarnitine; C18:1-OH = 3-hydroxyoctadecenoylcarnitine; C18-OH = 3-hydroxyoctadecanoylcarnitine; C6 = hexanoylcarnitine; C8 = octanoylcarnitine; C10:1 = decenoylcarnitine; C10 = decanoylcarnitine; C4-OH = 3-hydroxybutyrylcarnitine; C4 = butyryl/isobutyryl-carnitine.

**Table 2:** Confirmed diagnoses from December 2017 to April 2020.

Patient	Disorder	Cases
Newborns	Propionic Acidemia	1
	Glutaric Acidemia Type I	1
	Citrullinemia Type I	2
	Phenylketonuria	3
	Hyperphenylalaninemia	7
	Short Chain AcylCoA Dehydrogenase Deficiency	6
	Isovaleric Acidemia	1
	2-Metyilbutyryl-CoA Dehydrogenase Deficiency	1
	3-Methylcrotonyl-CoA Carboxylase Deficiency	3
Adults *	Citrullinemia Type I	1
Adults *	Short Chain AcylCoA Dehydrogenase Deficiency	5
	Vitamin B12 Deficiency	5

* all the adults affected patients were diagnosed through the screening of the correspond newborn.

**Table 3:** Newborns affected by short chain acyl-CoA dehydrogenase deficiency (SCADD).

Patients	Gender	C4 (I DBS)	C4 (II DBS)	EMA (Urine)	Mutation	Sympotms and Signs
case 1	M	1.08	0.52	↑	c.625G>A; c.320G>A compound heterozygous (p.Gly209Ser; Arg107His)	none
case 2	M	1.30	0.44	/	c.625G>A; c.136C>T compound heterozygous (p.Gly209Ser; p.Arg46Trp)	none
case 3	M	1.65	0.34	↑	c.625G>A homozygous (p.Gly209Ser)	none
case 4	M	0.95	0.40	↑↑	c.625G>A homozygous; c.1147C>T heterozygous (p.Gly209Ser; p.Arg383Cys)	none
case 5	M	0.95	0.51	↑↑	c.625G>A homozygous; c.1147C>T heterozygous (p.Gly209Ser; p.Arg383Cys)	none
case 6	M	0.94	0.24	↑	c.625G>A homozygous; c.366_367delC>T heterozygous (p.Gly209Ser; p.Tyr123Profs*24)	none

C4 = butyryl/isobutyryl-carnitine (cut-off = 0.62 M); DBS = dried blood spot; EMA = ethylmalonic acid (qualitative analysis, ↑ = low excretion, / = no excretion, ↑↑ = elevated excretion); M = male.

**Table 4:** Adult patients affected by SCADD, identified through newborn screening of their relatives babies.

Patients	Gender	C4 (I DBS)	EMA (Urine)	Mutation	Sympotms and Signs
case a	M	0.77	↑↑	c.625G>A homozygous; c.1147C>T heterozygous (p.Gly209Ser; p.Arg383Cys)	muscle weakness, urge of small and frequent meals,
case b	F	0.84	↑↑	c.625G>A homozygous; c.1147C>T heterozygous (p.Gly209Ser; p.Arg383Cys)	muscle weakness, urge of small and frequent meals,
case c	M	3.61	↑↑	c.625G>A homozygous; c.136C>T heterozygous (p.Gly209Ser; p.Arg46Trp)	muscle weakness, urge of small and frequent meals
case d	F	0.14	↑	c.625G>A; c.320G>A compound heterozygous (p.Gly209Ser; Arg107His)	none
case e	F	0.30	↑	c.625G>A; c.136C>T compound heterozygous (p.Gly209Ser; p.Arg46Trp)	none

C4 = butyryl/isobutyryl-carnitine (cut-off = 0.62 M); DBS = dried blood spot; EMA = ethylmalonic acid (qualitative analysis, ↑ = low excretion, ↑↑ = elevated excretion); M = male; F = female.
